# Self-Sterilizing Sputtered Films for Applications in Hospital Facilities

**DOI:** 10.3390/molecules22071074

**Published:** 2017-06-28

**Authors:** Sami Rtimi, Stefanos Giannakis, Cesar Pulgarin

**Affiliations:** Group of Advanced Oxidation Processes, Swiss Federal Institute of Technology, EPFL-SB-ISIC-GPAO, Station 6, CH-1015 Lausanne, Switzerland; stefanos.giannakis@epfl.ch

**Keywords:** antibacterial surfaces, light, metal oxides, coatings, magnetron sputtering

## Abstract

This review addresses the preparation of antibacterial 2D textile and thin polymer films and 3D surfaces like catheters for applications in hospital and health care facilities. The sputtering of films applying different levels of energy led to the deposition of metal/oxide/composite/films showing differentiated antibacterial kinetics and surface microstructure. The optimization of the film composition in regards to the antibacterial active component was carried out in each case to attain the fastest antibacterial kinetics, since this is essential when designing films avoiding biofilm formation (under light and in the dark). The antimicrobial performance of these sputtered films on *Staphylococcus aureus* (MRSA) and *Escherichia coli* (*E. coli*) were tested. A protecting effect of TiO_2_ was found for the release of Cu by the TiO_2_-Cu films compared to films sputtered by Cu only. The Cu-released during bacterial inactivation by TiO_2_-Cu was observed to be much lower compared to the films sputtered only by Cu. The FeOx-TiO_2_-PE films induced *E. coli* inactivation under solar or under visible light with a similar inactivation kinetics, confirming the predominant role of FeOx in these composite films. By up-to-date surface science techniques were used to characterize the surface properties of the sputtered films. A mechanism of bacteria inactivation is suggested for each particular film consistent with the experimental results found and compared with the literature.

## 1. Introduction

Hospital acquired infections (HAIs) are on the rise in Europe, infecting 5–7% of hospital patients staying beyond 10 days with the consequent high cost related to the long and costly hospital residence time [1]. Thirty years ago, Domek et al. [2] reported the inactivation of coliform bacteria by Cu, later Keevil’s group reported the Cu inactivation of *MRSA* [3] and *E. coli* [4]. The Cu-ions released from coated surfaces were reported to induce a strong biocidal effect below the cytotoxic levels accepted for mammalian cells. This consideration validates the use of Cu-immobilized devices in bloodstream infections [5]. The disinfection in some cases seems to proceed via an oligodynamic effect due to the low amounts (in the ppb/ppm range) of Cu or Ag released by these surfaces [6]. Cu-ions have been shown to complex proteins and break hydrogen bonds within the DNA opening the double helix [7].

Antimicrobial coatings are being investigated to prepare implants and medical devices [8,9,10,11]. Recently, research groups reported Chemical Vapor Deposition (CVD) of Cu-titania films being applied in single or multilayered coatings [8,9,10,11,12,13]. Innovative films against MRSA infections have been recently reported for packaging materials [14], plastics [15] and stethoscopes [16]. Boyce et al. [17] found MRSA contamination up to 65% in the hospital staff gloves and uniforms due to the contact in hospital-infected rooms/surfaces. Later, Bhalla et al. [18] showed that hospital workers frequently had infected gloves/uniforms in variable concentrations with the bacteria/fungi available in the hospital facilities. Bacteria invade, adhere and form biofilms that tightly glue to the surface (catheters or other medical devices) [19]. For this reason, biofilm formation has to be precluded. Catheters impregnated with antibiotics/antiseptics or both showed a short-term effect lasting only a few days. This is due to the rapid release of the antibacterial agent. This complicated in hospital settings by the increased bacterial resistance to many antibiotics affecting patients during long-hospital residence times [20,21].

Studies have shown rapid killing of bacterial cells when exposed to Cu-surfaces but the mechanism of the Cu-MRSA killing is still controversial [6,7,8,9,10,11,12,13]. The Cu-antimicrobial action seems to comprise the cellular metabolism damaging the cell DNA [22]. A recent study has shown that the uptake of Cu-ions by MRSA was fast and damaged the cell DNA, but the mechanism of this uptake remains controversial and more work is needed in this direction [1,22].

Recently, Heidenau et al. [10,11] demonstrated that in Ag-Cu amalgams, Cu in extremely low amounts inactivate more effectively bacteria compared to other metals. The Ag-Cu films were found to present surfaces with a high in vitro compatibility. Recent work in our laboratory with Cu-sputtered surfaces induced a faster kinetic bacterial inactivation [23,24] compared to Ag-sputtered surfaces [25,26]. TiO_2_ surfaces in the dark are ineffective against bacterial infection, but Cu-TiO_2_ surfaces introduce antibacterial action in the dark on medical implants [27,28]. TiO_2_ has been reported to increase the adhesion of Cu on glass and other surfaces avoiding leaching during disinfection and therefore assumes the role of a protective additive hindering the Cu-release.

In the past few years, the continuous exposure to antibiotics over long times has led to increased antibiotic resistance of bacteria. However, only a few pathogens display resistance to Ag and Cu and combinations thereof [1,6,29,30]. Cu-Ag films show long-operational lifetime, which is not the case for antibiotics/antiseptics rapidly detaching from the film surface. This is important when antibacterial films are used in connection to bloodstream. In this review, we focus on the preparation of innovative antibacterial coatings on 2D surfaces (polyethylene or polyurethane films) and on 3D complex shape medical devices (intravascular catheters). The sputtering of uniform, adhesive coatings with Cu and Ag on low thermal resistant fabrics like hospital textiles and thin polymer films are reviewed as well as material related to TiO_2_, TiO_2_-Cu (both on polyester), FeOx and FeOx-TiO_2_ (both on polyethylene films) [30,31,32,33,34,35].

## 2. Antibacterial Coatings: From Conventional to up to Date Methods

Conventional deposition techniques e.g., sol-gel or films prepared via the colloidal route enable the synthesis of materials with a high thermal resistance since a temperature of a few hundred degrees has to be applied to anneal the active antibacterial component to the particular substrate. Commercial sol-gel methods have reported TiO_2_ and other thin films on heat resistant substrates [35]. 

Photo-induced reactions by finely dispersed Ag-nanoparticles (Ag-NPs) of ~2 nm in size have been reported to inactivate *E. coli* and *Staphylococcus aureus*. This dispersion also showed photo-switchable behavior involving a transition from an initial hydrophobic surface towards a super-hydrophilic surface within the bacterial inactivation time under light irradiation. The contact angle (and surface energies) was followed under light and in the dark. The reversible process to reestablish the initial hydrophobicity (dark storage of the sample after bacterial inactivation under light) was seen to happen within long times (>24 h) [35].

Estimates for the effective bacteria reduction by Ag-coated surfaces suggest that these may be able to reduce the contamination in hospitals and public places [36,37,38,39]. The level found for these bacteria in many hospitals is higher than the allowed level for the healthcare rooms. For example, the contamination of 10^5^ CFU/cm^2^ was observed in a diabetic wound dressing. Nevertheless, near the patient, a microbial density of about 10^2^ CFU/cm^2^ was found. The use of catalytic/photocatalytic textiles (for beddings, curtains, lab-coats…) can drastically decrease the bacterial propagation [38,39,40,41,42].

The major limitations towards the use of sol-gel photoactive surfaces are related to (i) the lack of uniformity of these sol-gel films; (ii) the non-reproducible preparation observed from different batches with the same composition and (iii) the lack of mechanical stability and adhesion to the substrate [24,25,26,40]. The sputtering methods described in this review have been applied to obtain robust antibacterial films on cotton, polyester, polyethylene and polyurethane thin films [34,35,40].

During the last four decades, the sputtering of surfaces for industries in the aviation, car and machine tool sectors was used to protect the surfaces against corrosion by sputtering micrometer thick coatings of Cr-Fe to avoid the corrosion of the Fe/Fe_2_O_3_ layers. Nowadays, thin coatings are used for many purposes such as to avoid surface corrosion, to serve as anti-reflective and to acquire self-cleaning and self-sterilizing properties. Recently sputtering in the presence of O_2_ have been carried out to deposit thin metal oxides on non-heat resistant substrates like textiles at temperatures <130 °C by DCMS, DCPMS [41] and HIPIMS [42]. During the last decade, Kelly et al. has addressed the preparation of antibacterial thin films by magnetron sputtering (DCMS, DCMP and HIPIMS). They characterized the film surface properties and corrosion and correlated with the antibacterial activities [43,44,45,46]. Several recent studies report that the antibacterial activity of metal/oxide films is a function of the dispersion, size and thickness of the metal/oxide coatings [5,8,12,13,30,44,46,47,48,49,50]. Diverse thicknesses in the TiO_2_ coatings led to a complete different Cu-dispersion, Cu nanoparticulate size and bacterial inactivation kinetics as recently reported [12,29,40,49,51,58]. During the last years, we investigated the bactericidal activity on Ag, Cu, and other metal oxides sputtered on polymers and textile fabrics. The antibacterial kinetics, microstructure and surface properties have been reported in details [40,41,42,50,51,52,53].

## 3. Photocatalytic Coated Polyester Showing Duality in the *E. coli* and MRSA Inactivation under Actinic Indoor Light

Recently, some laboratories have reported the preparation of antibacterial Ag, Cu and TiO_2_ coatings on glass and polymer films by depositing the metal/oxides by CVD and sputtering techniques [8,29,30,32,44,45,46]. The direct current magnetron sputtering (DCMS) co-deposition of TiO_2_ and Cu films leading to uniform, adhesive and robust layers on polyester (PES) at temperatures not exceeding 120–130 °C has been reported recently [29,30]. There is a need for innovative active coatings showing a fast bacterial inactivation kinetics and a high adhesion to the substrate. In this way, the formation of toxic biofilms spreading bacteria/virus/fungi in hospital facilities leading to increased HAIs may be precluded.

Recent studies report the preparation of TiO_2_, Cu and TiO_2_/Cu films by sol-gel methods inducing significant photo-induced bacterial inactivation of films deposited on glass surfaces from Ti-chloride/ethyl acetate annealed at 500 °C [40,47,48,49,50]. Our laboratory has reported *Escherichia coli* (*E. coli*) inactivation on TiO_2_/Cu sequentially sputtered (deposited one after the other starting with an under layer of TiO_2_ then an upper layer of Cu/CuOx). Moreover, the co-sputtering of TiO_2_-Cu (simultaneous deposition) on textiles leading to *E. coli* and methicillin-resistant *Staphylococcus aureus* (MRSA) reduction was reported [43]. The later reports the differential effect of actinic and visible light (400–700 nm) on the bacterial reduction kinetics on *E. coli* as a MRSA.

**Figure 1:**
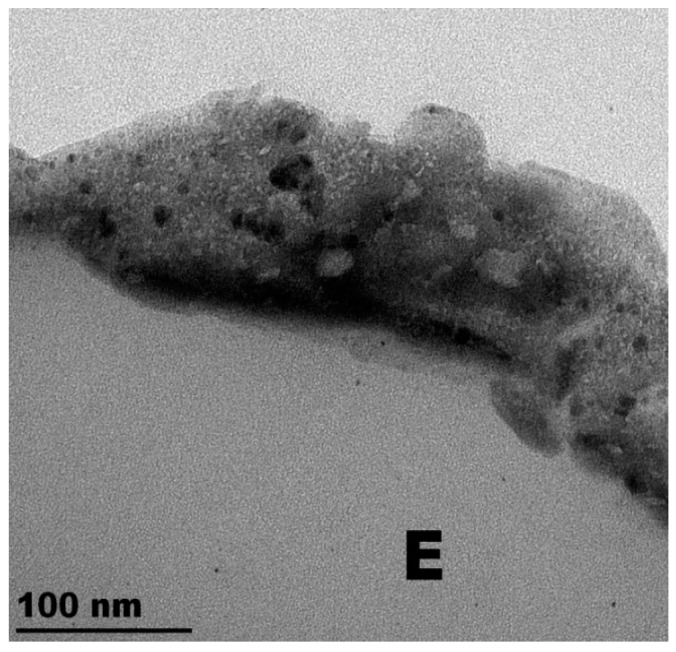
Transmission electron microscopy (TEM) of TiO_2_-Cu co-sputtered for 3 min on PES. “E” stands for the epoxide required to embed the sample during the sample preparation/cutting for the TEM image [32].

The TiO_2_-Cu-PES microstructure is shown by TEM in Figure 1. The denser Cu-clusters presented diameters between 16 and 20 nm while the TiO_2_ clusters presented smaller sizes from 5 and up to 10 nm. The TiO_2_-Cu coatings were 120 to 160 nm thick. This is equivalent to 500 to 800 atomic layers (being each layer 0.2 nm thick) each containing 10^15^ atoms/cm^2^ [51]. It showed close contact between the TiO_2_ and Cu-nanoparticles and was uniform in its microstructure [32,35,50]. 

**Figure 2:**
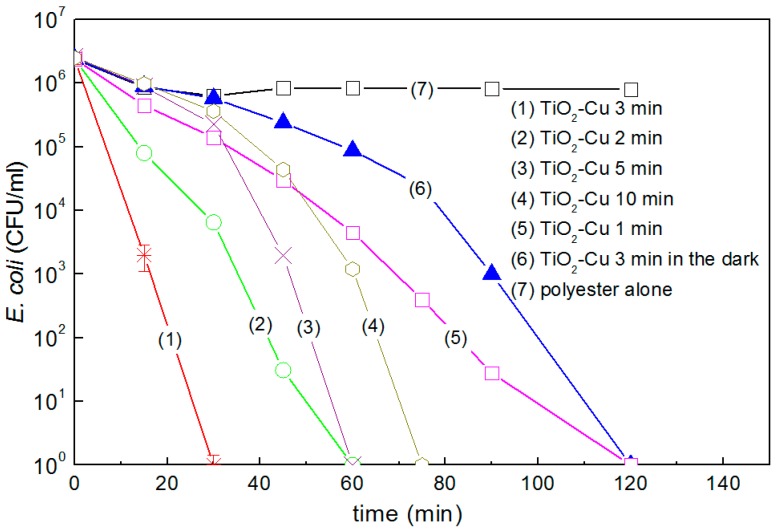
*E. coli* inactivation on TiO_2_-Cu co-sputtered for different times on PES as indicated in the traces: (1) 3 min, (2) 2 min, (3) 5 min, (4) 10 min, (5) 1 min (6) 3 min in the dark and (7) PES-alone. The bacterial reduction under light irradiation used a lamp Philips Master-18W/865 (4.65 mW/cm^2^), Error bars: standard deviation (*n* = 5%).

The bacterial inactivation by TiO_2_-Cu-PES under actinic light irradiation and in the dark was evaluated as a function of the amount of TiO_2_ and Cu sputtered on the substrate [41]. The deposition time of TiO_2_-Cu (co-sputtering) was optimized to determine the most suitable amount of TiO_2_ and Cu on the inactivating *E. coli.* In the dark, *E. coli* bacterial inactivation proceeds within 120 min on TiO_2_-Cu-PES. The mechanism of TiO_2_-Cu(CuO) mediated *E. coli* inactivation under light irradiation has been reported to involve interfacial charge transfer (IFCT) [29,40]. Bacterial reduction in the dark as shown in Figure 2, trace 6) and seems to proceed through a mechanism where O_2_ (air) reacts with the Cu^0^/Cu-ions. Figure 2, trace 1) shows the complete bacterial reduction under visible light within 30 min for TiO_2_-Cu samples co-sputtered for 3 min. Within 3 min, a sufficient amount of TiO_2_ and Cu was sputtered on the PES leading to the most suitable number of exposed catalytic sites leading to the fastest *E. coli* inactivation.

Co-sputtering for short times (1–2 min) did not deposit the necessary amount of TiO_2_ and Cu to induce a fast bacterial inactivation. Co-sputtering TiO_2_-Cu for 5 and 10 min (Figure 2) led to longer bacterial inactivation kinetics compared to TiO_2_-Cu (3 min). This is due to the inward charge diffusion of the generated charges in the TiO_2_ under band–gap irradiation [29,51]. In addition, longer sputtering times facilitate the TiO_2_ inter-particle growth decreasing the TiO_2_ contact surface with bacteria [52]. Figure 2, trace 6) shows complete bacterial reduction in the dark. Bacterial inactivation takes place in the dark possibly through an oligodynamic effect as recently reported by S. Rtimi [41,52,53]. The effect of Cu on bacteria has been associated to reactions blocking the of proteins/enzymes regulating the respiratory chain [32,41,52]. 

The electronic transfer between the TiO_2_/Cu and *E. coli* depends on the length of the charge diffusion in the TiO_2_/Cu layers. The diffusion of the charges induced by band-gap irradiation is a function of the TiO_2_ and Cu particle size and shape [54,55]. The interfacial distances between TiO_2_ and Cu/CuO on the polyester surface ranges from 5 nm and up. The IFCT, as shown in Figure 2, proceeds with a quantum efficiency depending on the light intensity and the nanoparticulate size [56,57,58]. Quantum size effects occur in particles with sizes ~10 nm and about 10^4^ atoms or smaller [59]. The surface composition and properties of the TiO_2_-CuO play a role in the charge transfer. The bacterial inactivation kinetics depends on the film (i) surface defects; (ii) surface imperfections; and (iii) dangling bonds on the edge of this composite. In TiO_2_-Cu composite, the charge recombination in nanoparticles is short due to their small particle size. The small particle size decreases the space for charge separation. In addition, the semiconductor space charge layer in both the TiO_2_ and CuO further decreases the potential depth available for the charge injection at the TiO_2_-Cu hetero-junction. This in turn, decreases the energy difference between TiO_2_ and Cu, which is not favorable for the charge injection [29,52]. The conduction band of CuO at −0.30 V vs. SCE (pH 7) is at a more negative potential than the potential required for one electron oxygen reduction [52]. Furthermore, the Cu^2+^ can also react with O_2_^−^:

O_2_ + H^+^ + e^−^ → HO_2_^•^ −0.22 V (1)

Cu^2+^ + O_2_^−^ → Cu^+^ + O_2_ (2)

**Figure 3:**
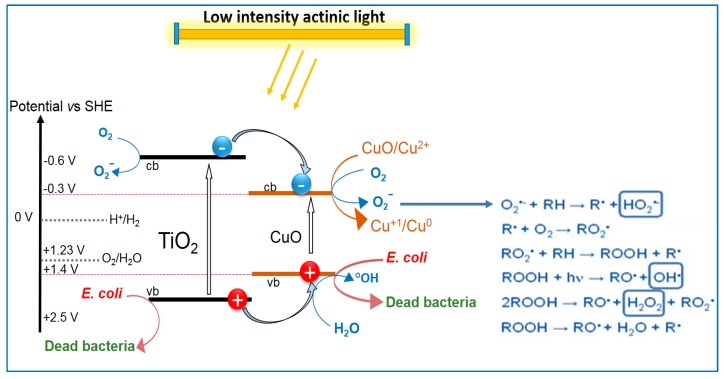
Diagram suggested for of bacterial inactivation under solar simulated light photocatalyzed by TiO_2_/Cu films sputtered on polyester (PES) [52].

The irradiation with solar simulated light induces the transfer of the e^−^ and h^+^ from TiO_2_ to CuO as shown in Figure 3 below. Charge transfer from ad-atoms to TiO_2_ under light has been investigated during the last few decades [40]. The potential energy levels of the TiO_2_ conduction band (cb) and TiO_2_ valence band (vb) lie above the CuO(cb) and CuO(vb) as shown in Figure 3. The partial recombination of e^−^/h^+^ in TiO_2_ is hindered by the charge injection into CuO. The interfacial charge transfer (IFCT) between the TiO_2_ and the CuO(vb) of +1.4 eV to the TiO_2_(vb) at 2.5 eV vs. SCE, pH 0, and proceeds with a considerable driving force due to the large potential energy difference between these two valence band levels.

A model for the charge transfer between TiO_2_ and CuO under solar light (UV-Vis) has been suggested [40,52] with respect to previous reports [56,57,58] and presented in Figure 3. The *E. coli* inactivation proceeded within a few minutes [29,52]. These TiO_2_(vb) holes react with the surface-OH groups of the TiO_2_ releasing OH-radicals. The CuO nanoparticles on the TiO_2_ can be reduced to Cu_2_O by the charges generated in the TiO_2_ under light and can later re-oxidize to CuO as investigated by X-ray photoelectron spectroscopy (XPS) in recent report [52]. The TiO_2_/Cu co-sputtered samples showed that TiO_2_ plays a stabilizing effect on the Cu-release from the co-sputtered surfaces during bacterial inactivation compared to Cu deposed individually [29,52]. A low amount of Cu-released in the ppb range inactivated both Gram-positive and Gram-negative bacteria, possibly through an oligodynamic effect as observed in dark runs but needing longer times [53,59].

## 4. Nanostructured Fe-Oxides for Self-Sterilizing through an Oligodynamic Effect: Surface Properties

Iron oxide nanoparticles (NP’s) are of considerable interest due to their wide applications in fields such as magnetic storage, medicine, chemical industries, catalytic materials and water purification [60]. The synthesis of Fe_2_O_3_, FeO and Fe_3_O_4_ involve routes including precipitation, sol-gel, hydrothermal, dry vapor deposition, has been carried out by way of micro-emulsion, electro-deposition and sonochemistry [61,62].

To avoid the time, work and reagents needed to separate the products from reactions catalyzed by suspensions at the end of water detoxification processes involving nanoparticles, metal/metal oxide coatings were prepared. Polymer-based films have been applied in protective coatings of medical devices [63,64], thin-films and bactericide/self-cleaning surfaces [64,65,66]. Films grafted by colloids weakly adhered to the substrate, have shown to be not entirely reproducible and can be wiped out of the polymer surface [49,66,67].

Polyethylene (PE) thin film is a flexible low cost polymer resistant to corrosion and withstands up to 120 °C for short times were coated/sputtered with FeOx [49,68]. Due to its low surface energy, the PE limits the adhesion of particles on its surface. In order to bind a higher amount of catalytic species on the PE surface suitable anchor groups have been used to attain acceptable catalysts loadings leading to the degradation of pollutants/bacteria occurring with a satisfactory kinetics. Surface pretreatment was necessary to increase the number of oxidative sites, hydrophilicity, and surface-roughness necessary for better FeOx bonding [69]. The polyethylene fabrics were pretreated in the cavity of an RF-plasma unit (13.56 MHz, 100 W, Harrick Corp., Ithaca, NY, USA) at a pressure of 1 torr. The topmost PE-layers of 2 nm (~10 atomic layers) were RF-plasma pretreated for 15 min. Oxygen RF-produced plasma reacts with the PE surface to induce groups like C-O, C=O, O-C=O, C-O-O- on the PE surface. This pretreatment introduces hydrophilic groups on the PE-surface and breaks the intermolecular PE- and the H-H bonds segmenting the PE-fibers [35,70]. The slightly positive FeOx binds the negatively charged pretreated PE (containing the overall negative carboxylic groups) through electrostatic interaction and chelation/complexation [68]. FeOx was sputtered from a target 5 cm diameter (Kurt Lesker, East Sussex, UK) positioned at 10 cm from the target by direct current magnetron sputtering (DCMS) on PE. The PE consists of highly branched low crystalline semi-transparent film with the formula H(CH_2_-CH_2_)_n_H (ET3112019, Goodfellow, Huntingdon, UK). The PE-FeOx mediated bacterial reduction was determined on *Escherichia coli* (*E. coli K12* ATCC23716) on 2 cm by 2 cm PE-FeOx samples under solar simulated light (52 mW/cm^2^, ~0.8 × 10^16^ photons/s) for utilization in environmental cleaning [68,71]. 

**Figure 4:**
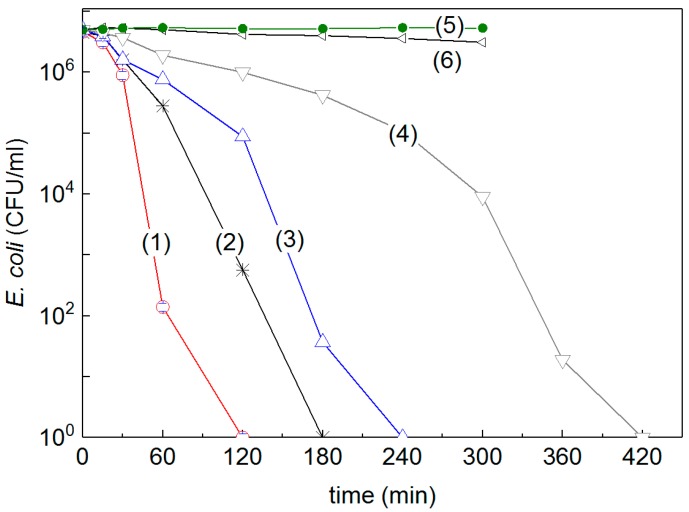
*E. coli* reduction on PE-FeOx pre-treated surfaces and sputtered for (1) 60 s; (2) 120 s; (3) 150 s; (4) 30 s and illuminated with solar simulated light of 52 mW/cm^2^; (5) PE-FeOx sputtered for 60 s in the dark and (6) Un-sputtered PE under solar simulated light; Error bars: standard deviation (*n* = 5%) [68].

Bacterial inactivation under low intensity solar simulated light on PE-FeOx sputtered films in Ar + O_2_ atmosphere proceeded with an acceptable kinetics [68]. The PE-FeOx films avoid the use of heavy metals whose discharge into the environment is not desired or admissible by pertinent sanitary regulations. The bacterial inactivation kinetics was attributed to the redox processes occurring on the surface. The regeneration of the surface initial catalytic states was reported to happen by simply washing the surface with NaOH. The PE-FeOx properties like surface polarity, roughness and stability were described in details [68,71]. Figure 4 presents the *E. coli* inactivation under solar simulated light irradiation for PE-samples pretreated with Rf-plasma and Fe-sputtered between 30 s and 150 s. It is readily seen that Fe-sputtering for 60 s (trace 1) led to the faster bacterial reduction time. The FeOx film thickness (42 nm equivalent to 210 atomic layers) led to the shortest bacterial reduction time [68]. If one atomic layer is ~0.2 nm thick and including 10^15^ atoms/cm^2^, the Fe deposition rate can be estimated as 3.5 × 10^15^ atoms/cm^2^ × s. Sputtering for 30 s (Figure 4, trace 4) did not deposit enough FeOx on the PE (0.040 wt % Fe_2_O_3_/weight PE). The longer bacterial reduction time shown in Figure 4, traces 4 and 5) for samples sputtered for 120 and 150s showing higher loadings > 0.084 wt % Fe_2_O_3_/weight PE. This was probably due to: (i) the increase in layer thickness leading to the bulk inward diffusion of the charge carriers [59,68], (ii) the increased size of the FeOx at longer sputtering times leading to cluster agglomeration [68,71] and (iii) the increase of the Fe-metal content with respect to FeOx. The bacterial reduction on PE-FeOx films does not proceed like on PE-alone under simulated solar irradiation.

PE-FeOx mediated bacterial reduction was investigated and remained stable up to five cycles (one cycle = one bacterial inactivation run + one water washing run) [68]. The Fe-leached out in ppb amounts during *E. coli* bacterial reduction was determined by inductively coupled mass spectrometry (ICP-MS) and showed a small release of Fe-ions to the environment at below toxicity levels. PE-FeOx induced stable re-cycling during bacterial inactivation trials [68]. Evidence of ppb amounts of Fe was observed in the PE-FeOx mediated bacterial inactivation suggesting an oligodynamic effect. Until now, only heavy meals as Ag, Pt and Pd have been reported to induce bacterial inactivation through the oligodynamic effect, but, this introduces undesirable/detrimental metals into the environment [53]. Fe_2_O_3_ colloids have been reported to leach consistent amounts of Fe during the degradation of pollutants in aqueous suspensions. Fe_2_O_3_ is a stable n-type semiconductor responding to visible light up to 500 nm with a band-gap of 2.2 eV able to separate the electrons at a potential ~0.1 eV and holes at ~2.3 eV as a function of the applied light (pH 6–7) [68].

By XPS analysis, the PE-FeOx surface atomic concentration and the changes in the Fe-oxidation states during the bacterial inactivation period were followed. The initial Fe(III) in Fe_2_O_3_ at 712.2 eV decreases from 80.0% (time zero) to 53.0% after the disinfection period. Fe in the form of Fe_3_O_4_ at 713.6 eV and Fe(II) with peaks at 709.7 eV was detected before the bacterial disinfection [68]. After disinfection, Fe(III), FeO(II/III) and Fe(II) peaks were: 711.4, 708.6 and 713.8 eV, respectively. These changes point out to the shift in the oxidation states in the Fe-oxides during bacterial reduction. The catalysis deriving the bacterial reduction contains three different FeO_x_ oxide species each one offering a different potential couple and its own surface potential (eigenvalue). The high turnover of the biological material on the photocatalyst surface avoids the accumulation of residual intermediates during bacterial inactivation as observed for the lack of C1s and N2p peaks after the bacterial inactivation on the PE-FeOx surface [68,70].

The electrostatic attraction between the bacteria and PE-FeOx is a dominant effect at distances below four Angstroms, polarizing strongly the interaction between the PE-FeOx within this short distance. A mechanism of reaction was recently suggested taking into consideration the bacterial inactivation dynamics and Equations (3)–(6) below. The generation of highly oxidative radicals (ROS) by the O_2_ (air) reduction under the solar simulated light was observed concomitantly to the Fe(III)/Fe(II) reduction during bacterial oxidation leading to CO_2_ and a small amount of mineral trace residues:

(3) $${\mathrm{Fe}}_{2}O_{3}\left({\mathrm{Fe}}^{2+}\right)+O_{2}\;(\mathrm{air})\;+H^{+}\to {\mathrm{Fe}}_{2}O_{3}\left({\mathrm{Fe}}^{3+}\right)+{{\mathrm{HO}}_{2}}^{•}$$

The HO_2_^•^ radical would convert to O_2_^−^ at the biocompatible pH 6–7 through Equation (4):

(4) $${{\mathrm{HO}}_{2}}^{•}\;\Leftrightarrow \;H^{+}\;+\;{O_{2}}^{-}\;\mathrm{pKa}=4.8$$

In the presence of Fe-ions, the HO_2_^•^ decomposes at much slower kinetics compared to the fast reaction between O_2_^−^ + Fe^3+^, it is suggested to follow reactions (5) and (6) below:

(5) $${{\mathrm{HO}}_{2}}^{•}\;+\;{\mathrm{Fe}}^{3+}\;\to \;{\mathrm{Fe}}^{2+}+\;H^{+}\;+\;O_{2}\;3.3\times 10^{5}\;M^{-1}s^{-1}$$

(6) $$O^{2-}\;+\;{\mathrm{Fe}}^{3+}\;\to \;O_{2}\;+\;{\mathrm{Fe}}^{2+}\;1.0\times 10^{9}\;M^{-1}s^{-1}$$

The reaction between PE-FeOx and the bacteria cell wall involves diffusion of the metal-ions and absorption/translocation of the metal-ions on the bacterial cell bilayer. Electrostatic and Van der Waals interactions and controlled diffusion of FeOx at the interface with *E. coli* cells drive the interaction between the bacteria and the catalyst/photocatalyst surfaces [68].

## 5. Coupling of TiO_2_ and Fe-Oxide: Innovative Preparation for Self-Sterilizing Surfaces

Binary-oxides semiconductors due to their optical absorption and semiconductor behavior have been widely used for environmental decontamination purposes like FeOx-TiO_2_. These binary oxides play an important role in pollutants and bacterial abatement involving redox processes [72,73]. Supported photocatalyst films, for self-cleaning and self-sterilizing purposes have recently been developed. This works involves the grafting of narrow band-gap semiconductors increase the light absorption in the visible region [68,72,73,74,75,76]. Work of this kind involves the selection of the meta/oxide components taking into consideration factors like (i) the surface spectral properties; (ii) the transients generated under femto-second laser pulses induced in the visible light region (545 nm /25 fs); (iii) the bacterial inactivation kinetics of *E. coli* under low intensity solar/visible light and effect of different irradiation intensities and (iv) the mechanism of electron transfer from the FeOx used as photosensitizer to the low lying TiO_2_ trapping states as recently reported [73].

**Figure 5:**
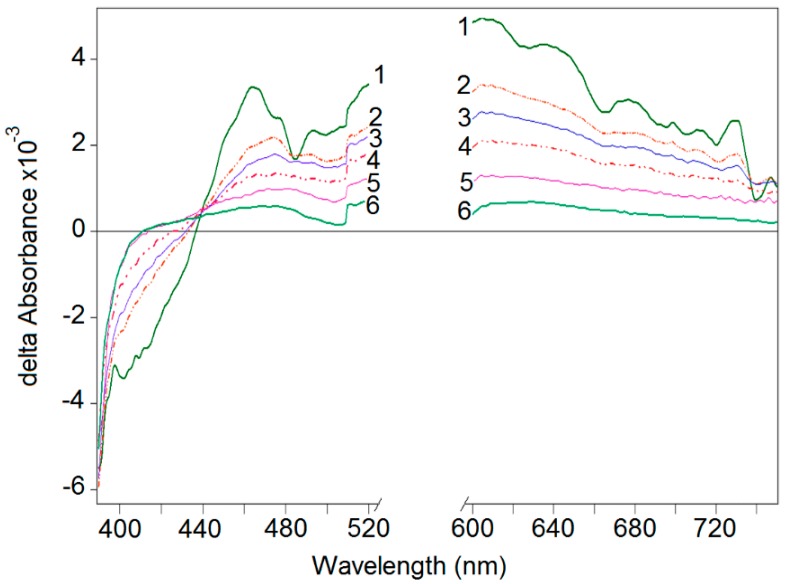
Transient spectra of the FeOx-TiO_2_-PE as a function of wavelength after femtosecond laser pulse 25 fs (544 nm). Time delays: (1) 150 fs; (2) 500 fs; (3) 1 ps; (4) 3 ps; (5) 10 ps; (6) 500 ps [73].

Femto-second ultra-fast kinetics is a powerful tool to detect and register the initial charge separation within very short times when PE-FeOx-TiO_2_ is irradiated under visible light [8]. Femto-second kinetics pulses in the visible range (545 nm/25 fs) to photo-induce and identify the short-lived transients. These transients are shown in Figure 5 at different pulse delay times as a function of wavelength [73,75]. FeOx absorbs laser pulses in the visible leading to the separation of the cb (e−) and vb(h+) or excited d-d states. Figure 5 also shows that the spectral features are about the same for different delays. An increase in the pulse delay up to 500 ps, leads to a decrease in absorption bands. Additional experiments showed that the main part of the absorption amplitude at 600 nm decays within 25 ps (t_½_~25 ps). Mid-gap Fe d-d states have been suggested as the main trapping sites with lifetimes of a few hundred picoseconds. The d-d states are ascribed to local excitons in the FeOx matrix [73]. In Figure 5, the electron trapping process was initiated at times of 150 fs and compete with electron-hole recombination. The absorption band with a maximum at 600 nm was assigned to the cb(e−) spectrum of the FeOx-TiO_2_ i.e., an IFCT process occurring at the heterojunction [75,76]. These ultra short-lived transients in the visible region lead later to longer-lived intermediates in the minute range able to inactivate bacteria under band-gap continuous irradiation [73,74,75].

## 6. Conclusions

The preparation of TiO_2_, TiO_2_-TiO_2_, FeOx and FeOx-TiO_2_ thin films and their dynamics have been reviewed. The bacterial interactions with the film surface in the dark and under light conditions were discussed. The reactivity of TiO_2_-Cu towards bacteria seems to proceed through mechanisms that are still controversial. TiO_2_-Cu films obtained by sputtering were described as well as their surface properties. The mechanism of bacterial inactivation by Cu, TiO_2_ and FeOx possibly involve an oligodynamic effect. Innovative PE-FeOx composites/coatings may have a potential application in disinfection for biomedical devices favored by the low Fe-cytotoxicity and high Fe-biocompatibility compared to heavy metals like Ag, Pt, and Au. The co-sputtered FeOx-TiO_2_-PE films show also a potential to improve the removal of pathogens and prevent biofilm formation under sun/visible light.
